# Two Distinct Pathways of B-Cell Development in Peyer’s Patches

**DOI:** 10.1155/1995/46974

**Published:** 1995

**Authors:** Philip J. Griebel, Birgit Kugelberg, Giorgio Ferrari

**Affiliations:** 1 Basel Institute for Immunology 487-Grenzacherstrasse Postfach Basel CH-4005 Swaziland; 2 Veterinary Infectious Disease Organisation 120 Veterinary Road University of Saskatchewan Saskatchewan Saskatoon S7N 5E3 Canada

**Keywords:** B lymphopoiesis, CD5+ B cells, corticosteroid, Peyer's patch, sheep, thymus

## Abstract

The developmental biology of sheep ileal and jejunal Peyer’s patches (PP) was investigated using corticosteroids to deplete immature B lymphocytes. During a 7-day treatment with dexamethasone, ileal PP follicular (iPf)B-cell proliferation was arrested and most iPfB-cells died. This resulted in follicular involution with the survival of mesenchymal cells. No iPfB-cell proliferation was detected in follicular remnants for 4 weeks postdexamethasone treatment, and during a subsequent 3-month period, there was limited iPfB-cell proliferation that resulted in a partial regeneration of follicles. Ileal PP involution was also associated with a severe B lymphopenia that persisted for over 14 weeks and was characterized by the survival of primarily isotype-switched and CD5^+^ sIgM^+^ B-cells in blood. In contrast, the size of jejunal PP follicles was reduced following dexamethasone treatment, but intrafollicular B-cell proliferation was not arrested. Furthermore, within 4 weeks, the jejunal PP follicles had recovered in size and cellularity and there was no disruption in IgA plasma-cell production. Thus, dexamethasone selectively depleted iPfB-cells and revealed that the ileal and jejunal PPs contain functionally distinct B-cell populations. The partial regeneration of the iPfB-cell population indicated that either an intrafollicular, corticosteroid-resistant B-stem cell existed or that ileal PP follicles can be repopulated by circulating B-cells. Finally, the association between ileal PP involution and the absence of circulating, CD5^-^ B^-^cells confirmed that this lymphoid tissue provides an essential environment for conventional sIgM^+^ B-cell development.


*Developmental Immunology*, 1996, Vol 4, pp. 263–277
Reprints available directly from the publisher
Photocopying permitted by license only

© 1996 OPA (Overseas Publishers Association)
Amsterdam B.V. Published in The Netherlands
by Harwood Academic Publishers GmbH
Printed in Singapore

### Two Distinct Pathways of B-Cell Development in Peyer's Patches

PHILIP J. GRIEBEL,* BIRGIT KUGELBERG, and GIORGIO FERRARI

*Basel Institute for Immunology, 487-Grenzacherstrasse, Postfach, CH-4005, Basel, Switzerland*

The developmental biology of sheep ileal and jejunal Peyer's patches (PP) was investigated using corticosteroids to deplete immature B lymphocytes. During a 7-day treatment with dexamethasone, ileal PP follicular (iPf)B-cell proliferation was arrested and most iPfB-cells died. This resulted in follicular involution with the survival of mesenchymal cells. No iPfB-cell proliferation was detected in follicular remnants for 4 weeks postdexamethasone treatment, and during a subsequent 3-month period, there was limited iPfB-cell proliferation that resulted in a partial regeneration of follicles. Ileal PP involution was also associated with a severe B lymphopenia that persisted for over 14 weeks and was characterized by the survival of primarily isotype-switched and $CD5^+sIgM^+$ B-cells in blood. In contrast, the size of jejunal PP follicles was reduced following dexamethasone treatment, but intrafollicular B-cell proliferation was not arrested. Furthermore, within 4 weeks, the jejunal PP follicles had recovered in size and cellularity and there was no disruption in IgA plasma-cell production. Thus, dexamethasone selectively depleted iPfB-cells and revealed that the ileal and jejunal PPs contain functionally distinct B-cell populations. The partial regeneration of the iPfB-cell population indicated that either an intrafollicular, corticosteroid-resistant B-stem cell existed or that ileal PP follicles can be repopulated by circulating B-cells. Finally, the association between ileal PP involution and the absence of circulating, $CD5^-$ B-cells confirmed that this lymphoid tissue provides an essential environment for conventional $sIgM^+$ B-cell development.

KEYWORDS: B lymphopoiesis, $CD5^+$ B cells, corticosteroid, Peyer's patch, sheep, thymus.

### INTRODUCTION

Investigations in rodents have established the concept that after birth, the bone marrow functions as the primary site of B-cell generation (Claman et al., 1966; Mitchell and Miller, 1968; Osmond, 1980), and investigations in both rodents and rabbits revealed that the Peyer's patches (PPs) function as a secondary, antigen-dependent lymphoid tissue that plays a major role in mucosal immunity (Pollard and Sharon, 1970; Craig and Cebra, 1971; Husband and Gowans, 1978). Consistent with this hypothesis, lymphoid follicles in the PPs develop postnatally in these species (Hummel, 1935; Waksman et al., 1973; Abe and Ito, 1977). However, in humans (Cornes, 1965), sheep (Reynolds and Morris, 1983), pigs (Chapman et al., 1974), and other species (Carlens, 1928), the lymphoid follicles of the PPs are well-developed prior to birth, and in PPs of fetal sheep, there is a high level of B-cell proliferation (Reynolds and Morris, 1983). This fetal developmental suggests that B-cell development in the PPs is, to some extent, antigen-independent and T-cell-independent. Thus, PPs may function as a site of both antigen-dependent and antigen-independent B-cell development.

Located along the small intestine of sheep are two distinct types of PPs: the ileal PP and the jejunal PP. These two PPs differ markedly in their life history, histology, lymphocyte composition, B-cell differentiation, and their role in the development of the humoral immune system. During ontogeny, lymphoid follicles develop first in the jejunal PPs, but in

*Corresponding author. Present address: Veterinary Infectious Disease Organisation, 120 Veterinary Road, University of Saskatchewan, Saskatoon, Saskatchewan, Canada S7N 5E3.

late-term fetuses, the follicular B-cells proliferate at a high level in both PPs (Reynolds and Morris, 1983). However, B-cell development in the two PPs appears to diverge at birth (Griebel et al., 1992). Jejunal PPs are active throughout the life of the animal and are a site for T-cell-dependent antigen responses with the production of both IgG1 and IgA PCs (Gerber et al., 1986; Griebel and Ferrari, 1995). In contrast, in the ileal PP, the production of B-cells (Griebel and Ferrari, 1994) and diversification of the immunoglobulin (Ig) receptor repertoire (Reynaud et al., 1995) are antigen-independent and T-cell-independent and the lymphoid follicles involute following sexual maturity (Reynolds and Morris, 1983). In young sheep, the ileal PP is the primary source of B-cells for all lymphoid tissues and provides an essentials environment for B-cell development (Gerber et al., 1986; Reynolds et al., 1991).

Immature lymphocytes, such as cortical thymocytes (Ishidate and Metcalf, 1963) and B-cells in lymphoid follicles of the chicken bursa of Fabricius (Glick, 1957), display a high corticosteroid sensitivity. In contrast, mature lymphocyte populations in secondary lymphoid tissues are much less sensitive to the cytolytic effects of corticosteroid (Cupps and Fauci, 1982). The sheep ileal PP B-cells display many of the characteristics of a primary lymphoid tissue and should display a high corticosteroid sensitivity relative to other gut-associated lymphoid tissues. Previous analyses of the V-J junctional diversity of rearranged λ Ig light-chain (LC) genes suggested that the B-cell population in each ileal PP follicle was oligoclonal, arising from a limited number of B-cell immigrants during fetal development (Reynaud et al., 1991). *In vivo* (Reynolds, 1986) and *in vitro* (Griebel and Ferrari, 1994) analyses of iPfB-cell production and death further support the idea that a closed population of iPfB-cells is maintained by self-renewing proliferation. Therefore, it was postulated that if corticosteroid treatment induced B-cell death and arrested proliferation, then follicular involution should follow. A dexamethasone-treatment protocol was developed that induced thymic involution and arrested B lymphopoiesis in the ileal PP of young sheep. The consequences of dexamethasone treatment were then compared for the ileal and jejunal PPs that contain functionally and phenotypically distinct B-cell populations (Hein et al., 1989; Griebel and Ferrari, 1995).

### MATERIAL AND METHODS

### Reagents

Dexamethasone (9α-fluoro-16α-methylprednisolone) was purchased from Sigma (St. Louis); 5-bromo-2-deoxyuridine (BrdU) and mouse monoclonal (mAb) anti-BrdU (BMC 9318) were purchased from Boehringer-Mannheim (Mannheim, Germany). The biotinylated-, fluorescein isothiocyanate (FITC-), and phycoerythrin (PE-) conjugated, isotype-specific goat anti-mouse Ig reagents were purchased from Southern Biotechnology (Birmingham, AL). The PE-conjugated rat anti-mouse CD8α was purchased from Caltag (San Francisco). Soluble, recombinant fusion protein of murine CD40L-CD8α (mCD40L-CD8α; Lane et al., 1993) was a generous gift from Peter Lane (Basel Institute for Immunology, Basel, Switzerland) and was previously shown to react with sheep CD40 (Griebel and Ferrari, 1995). The anti-IgM (PIg45A), anti-IgG1 (Blg715A), anti-IgA (Blg312D3), anti-λ Ig LC (Blg501E), and anti-κ Ig LC (Blg43) mAbs were purchased from VMRD Inc. (Pullman, WA). The pan-B cell (Du2-104; Press et al., 1993), anti-CD4 (17D-13; Maddox et al., 1985), anti-CD5 (ST1a; Beya et al., 1986), anti-CD8 (E95; Ezaki et al., 1987), anti-γδ T-cell receptor (127; Mackay et al., 1989), anti-CD44 (25-32; Mackay et al., 1988), and anti-CD45R (Mackay et al., 1987) mAbs were obtained from hybridomas maintained at the Basel Institute for Immunology, Basel. The anti-vimentin (VIM13.2) mAb was purchased from Sigma and the rabbit anti-human CD3 was purchased from Dakopatts (Glostrup, Denmark).

### Animals and Dexamethasone Treatment

All experiments were conducted using 4–5-week-old suckling lambs or 144-day gestation (148-day gestation period) fetuses, of either sex (Versuchbetrieb Sennweid, Olsburg, Switzerland). Dexamethasone was dissolved in dimethysulfoxide (Fulka Chemie, Fuchs, Switzerland) and prior to intravenous (iv) injection diluted to a final concentration of 1 mg/ml in 37°C phosphate-buffered saline (PBS). Six groups of lambs were used for dexamethasone treatment: 3 lambs were used to investigate the response of ileal PP follicles and the thymus to 0.02, 0.2, and 2.0 mg dexamethasone/kg body weight (BW) injected for 3 consecutive days; 3 lambs were used to evaluate the response of ileal PP follicles and the thymus to dexamethasone following daily injections of 2 mg/kg BW for 3, 5, or 7 days; 4 groups of 4 lambs were used to evaluate the responses of the PPs, thymus, and blood lymphocyte populations following daily injections of 2 mg/kg BW dexamethasone for 7 consecutive days. One group of 4 lambs was used to study the responses of lymphoid tissues during each of the following posttreatment intervals: days 1–10; days 1–28; days 1–52; and days 1–98.

### Tissue Collection, Cell Isolation, Immunohistochemistry (IHC), and Flow Cytometry

Blood collected in EDTA was used to determine total white cell counts, differential counts of leukocytes, and to isolate mononuclear cells with a discontinuous Percoll gradient (Griebel and Ferrari, 1995). Cell suspensions were prepared from lymphoid follicles of the PP and other tissues as described previously (Griebel and Ferrari, 1995; Griebel et al., 1994). Tissues for histology were first fixed in phosphate-buffered formaldehyde (12%) prepared in methanol and then dehydrated in graded ethanol before embedding in Technovit 7100 medium (Heraeus Kulzer, Wehrheim, Germany). Tissue sections, 1–1.5 µm thick, were mounted on precleaned glass slides, heated at 70°C for 1 hr and then stained for 3 min with 1% threonine-acetate (Fluka) prepared in distilled $H_2O$. Tissues for IHC were placed in cryomolds (Tissue-Tek II; Lab-Tek Products, Nunc Inc., Naperville, IL) and mucosal surfaces were covered with a thin slice of liver before embedding in O.C.T. compound (Miles Lab. Inc., Naperville, IL) and freezing on dry ice. The methods for indirect labeling of cell suspensions for flow cytometric analysis (FACScan; Becton Dickinson, Mountain View, CA), cell sorting (FACStar Plus, Becton Dickinson), and indirect immunoperoxidase staining of frozen tissue sections have previously been described in detail (Griebel et al., 1994; Griebel and Ferrari, 1995). To quantitate lymphocyte subpopulations in blood, the total number of blood mononuclear cells/ml blood was multiplied by the percent mononuclear cells labeled by the appropriate mAb and detected with flow cytometric analyses.

### BrdU Incorporation and Detection

BrdU was dissolved in 60°C PBS, cooled to room temperature, and injected iv at a final concentration of 20 mg/kg BW 30 min prior to collecting tissues. This procedure resulted in a detectable level of BrdU incorporation in 40–45% iPfB-cells and 8–10% of thymocytes (Griebel and Ferrari, 1995). Immunoperoxidase detection of BrdU incorporated in tissue sections was performed as previously described (Griebel and Ferrari, 1994).

### RESULTS

### Dexamethasone-Induced Involution of Primary Lymphoid Tissues

Preliminary experiments were completed to determine if dexamethasone induced involution of primary lymphoid tissues in young lambs. The thymus was used as a control organ because of its well-characterized corticosteroid sensitivity in mice (Ishidate and Metcalf, 1963; Clamen et al., 1971). The effect of dexamethasone treatment on the thymus and ileal PP was first evaluated with 0.02, 0.2, and 2 mg dexamethasone/kg BW administered for 3 consecutive days. Ileal PP histology and thymic weights were evaluated with tissues collected 24 hr after the last treatment. A marked reduction in thymic weight (40–60% decrease) and ileal PP follicular size and cellularity was observed at all doses of dexamethasone, but with 2 mg/kg, few follicular B cells were seen on tissue sections (data not shown). Three lamb were then injected with 2 mg dexamethasone/kg BW for 3, 5, and 7 days, and tissues were collected 24 hr after each treatment and 30 min after injecting BrdU. Few $BrdU^+$ cells were detected in ileal PP follicles following dexamethasone treatment for 3 days, and no detectable BrdU incorporation was observed following the 7-day treatment (data not shown). Thus, a 7-day treatment with 2 mg dexamethasone/kg BW was chosen to study the long-term effects of arrested iPfB-cell proliferation. This dexamethasone treatment regime resulted in a marked reduction in thymic cortex with a relative increase in the medullary region (Fig. 1b), but did not arrest proliferation of cortical thymocytes (Fig. 2b). Thymic weights for untreated, age-matched lambs were 58.6 + 7.2 g (mean + S.D. of values from 5 lambs), but during the first 2 weeks postdexamethasone, the average thymic weights were 12.8 + 3.2 g ($n$ = 5 lambs). The effects of dexamethasone on thymic architecture and thymocyte proliferation were no longer evident 4–5 weeks posttreatment (Fig. 2c).

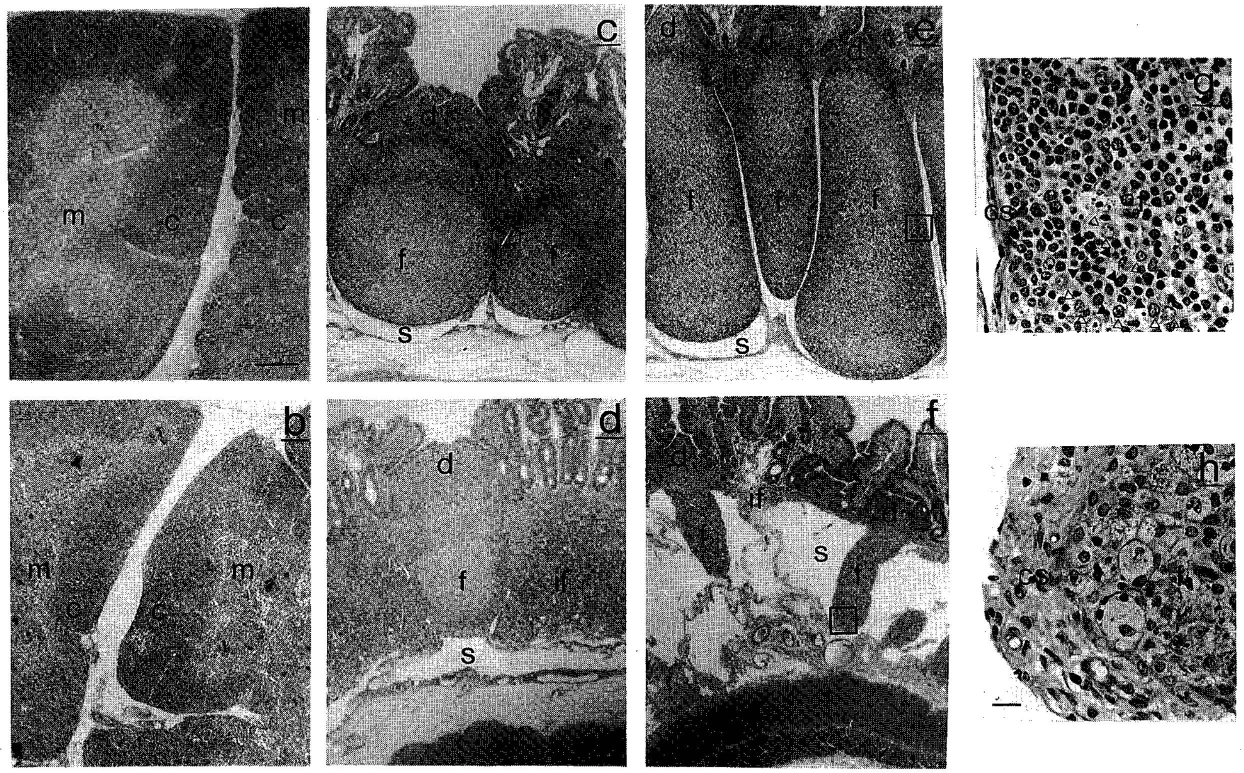

FIGURE 1. The histology of the thymus, jejunal PP, and ileal PP are compared for age-matched untreated (control) lambs and lambs treated for 7 days with 2 mg dexamethasone/kg BW (dex). Thymus: *a* (control) and *b* (dex) with the cortex (c) and medulla (m) indicated. Jejunal PP: *c* (control) and *d* (dex) with the lymphoid follicles (f), dome region (d), interfollicular area (if), and lymphatic sinuses (s) indicated. Ileal PP: *e* (control) and *f* (dex) with lymphoid follicles (f), dome region (d), interfollicular area (if), and lymphatic sinuses (s) indicated. Ileal PP follicle: *g* (control) and *h* (dex) with the follicular capsule (cs) and "nests" of lymphocytes (closed triangles) distributed among nonlymphoid stromal cells (open triangles) indicated. Magnification: *a–f* (bar [in *a*] = 300 μm); *g* and *h* (bar [in *h*] = 20 μm).

### Depletion of Ileal and Jejunal PP Follicles

Normal ileal PP follicles are enclosed by a thin fibrous capsule (2–3 cells thick), and in the outer follicle, there are numerous "nests" of lymphocytes distributed among nonlymphoid stromal cells (Fig. 1g). The majorityof lymphocytes in the outer follicle incorporate BrdU during a 30-min period (Fig. 2g). Dexamethasone treatment for 7 days resulted in a dramatic decrease in ileal PP follicular size (>90%) that was also associated with a reduction in the size and cellularity of the dome and interfollicular regions (Fig. 1f). Follicular remnants were suspended within dilated lymphatic sinuses (Fig. 1f) and no cell proliferation could be detected in the follicular remnants following BrdU injection (Fig. 2h). The capsule of involuted follicles was thickened and disorganized, lymphocytes were absent within the follicle, but there were many stromal cells with large nuclei and abundant, vacuolated cytoplasm (Fig. 1h). IHC confirmed that most cells within the follicular remnants were mesenchymal cells (Fig. 3a), including numerous macrophages (Fig. 3b). A small number of $CD4^+$ T-cells (Fig. 3c) and $CD45R^+$ cells (Fig. 3d) were also detected within follicular remnants. The IHC staining for $sIgM^+$ B-cells was difficult to interpret because an intense reticular staining pattern, suggestive of extracellular Ig, was observed within follicular remnants. Dexamethasone treatment also reduced the size and cellularity of the jejunal PP follicles but did not deplete intrafollicular lymphocytes (Fig. 1d) and did not arrest the proliferation of these cells (Fig. 2e). Thus, dexamethasone treatment selectively depleted most iPfB-cells, but many stromal cells, macrophages, and some $CD4^+$ T-cells survived.

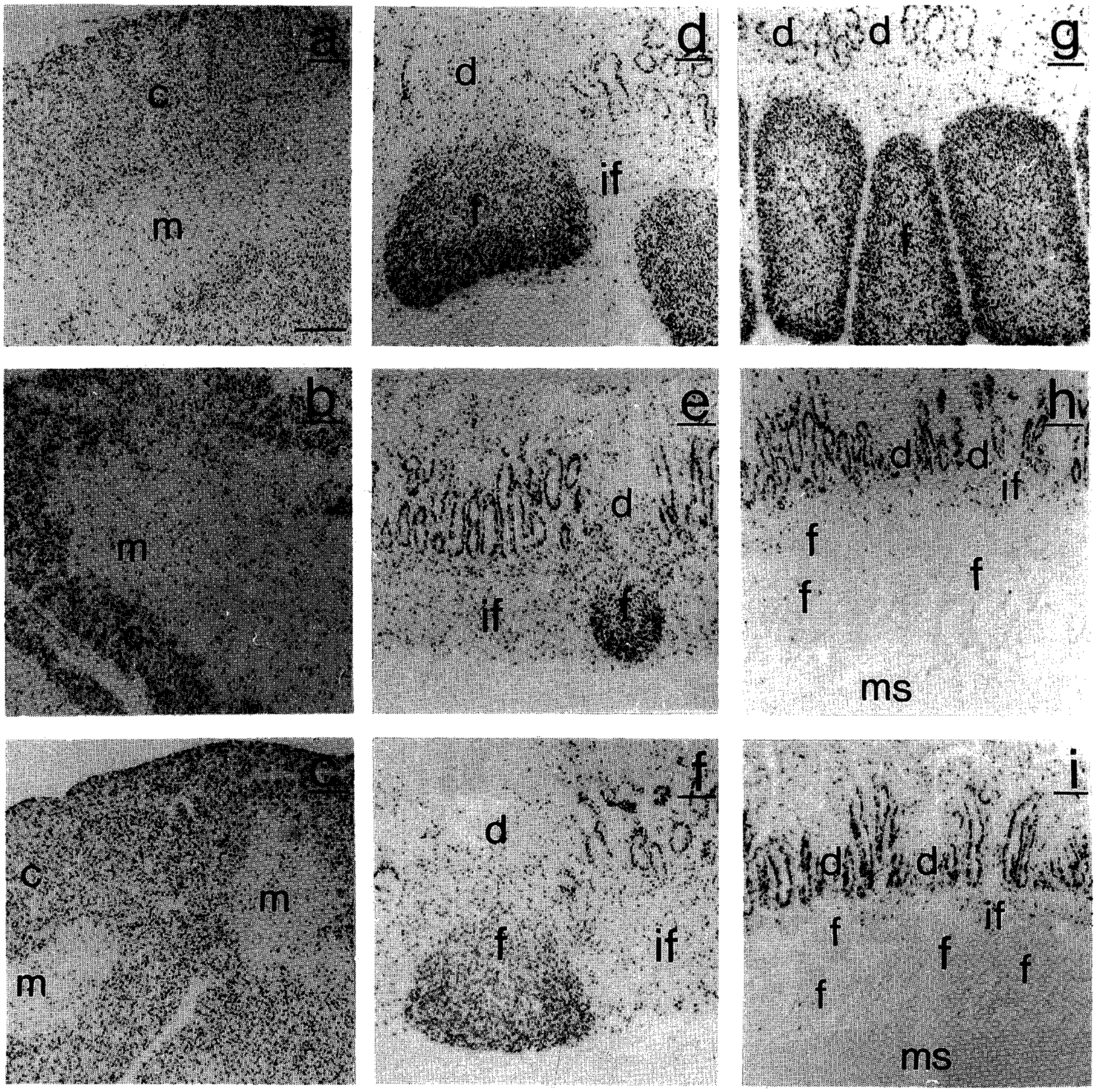

FIGURE 2. Cell proliferation, detected by BrdU incorporation, was analyzed in the thymus, jejunal PP, and ileal PP for age-matched, untreated lambs (control) and for lambs 1 day (D.1—postdex) and 3 weeks (D.21—postdex) after treatment for 7 days with 2 mg dexamethasone/kg BW. Thymus: *a* (control), *b* (D.1—postdex), and *c* (D.21—postdex) with the cortex (c) and medulla (m) indicated. BrdU incorporation was evident in the cortex both before and after dexamethasone treatment. Jejunal PP: *d* (control) and *e* (D.1—postdex), and *f* (D.21—postdex) with the lymphoid follicles (f), dome region (d), and interfollicular area (if) indicated. BrdU incorporation was prominent in the crypt epithelium and lymphoid follicles both before and after dexamethasone treatment. Ileal PP: *g* (control), *h* (D.1—postdex), and *i* (D.21—postdex) with lymphoid follicles (f) dome region (d), interfollicular area (if), and muscularis (ms) indicated. No BrdU incorporation was detected within the follicles following dexamethasone treatment, but BrdU incorporation in the crypt epithelium remained unchanged. Magnification: *a*–*i* (bar [in *a*] = 350 μm).

### Differential Regeneration of Ileal and Jejunal PP Follicles

Ileal PP follicles are characterized by a high mitotic rate, 15–20 × greater than that of the thymus (Reynolds, 1987). This B-cell proliferation occurs primarily in the outer follicle with few cells proliferating in the central follicle, dome region, or interfollicular region (Fig. 2g). Thus, BrdU incorporation

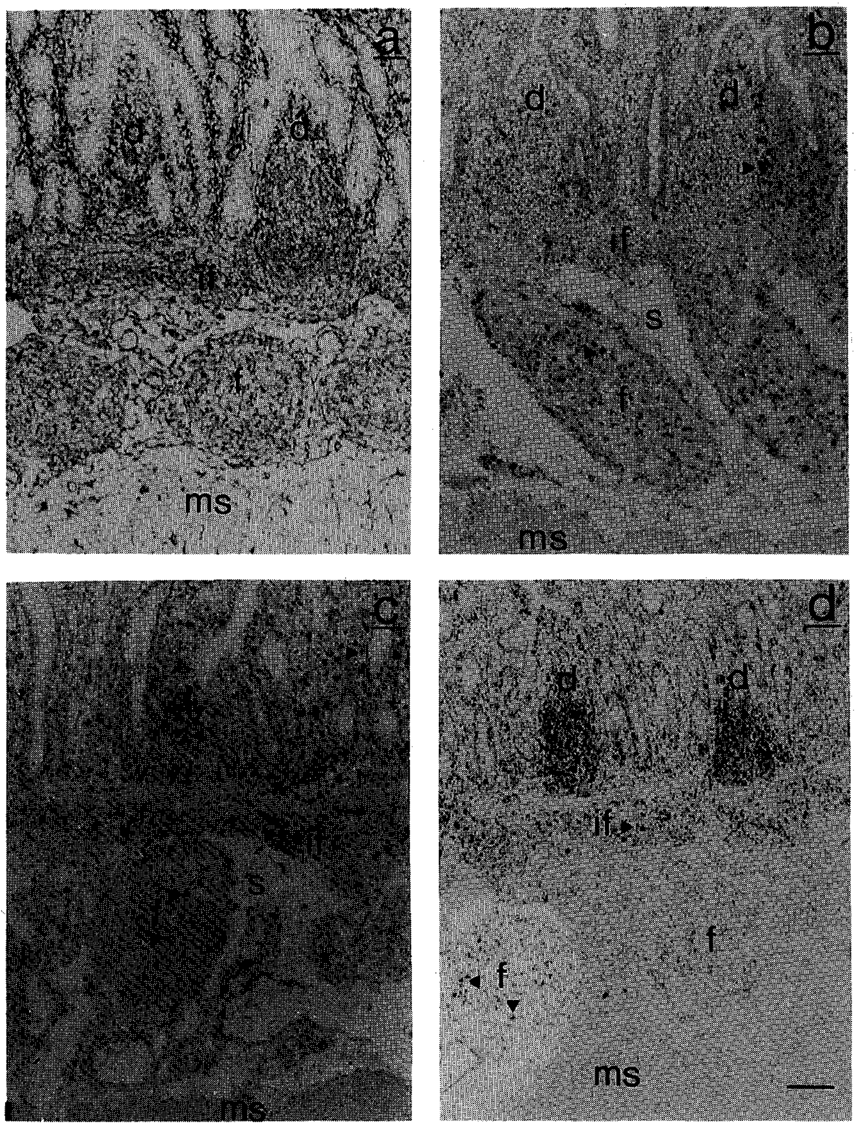

FIGURE 3. Immunohistochemical characterization of cells in ileal PP lymphoid follicles following treatment with 2 mg dexamethasone/kg BW for 7 days. *a*: The stain for mesenchymal cells (vimentin) revealed a network of cells in the lamina propria of the villi, numerous cells within the dome (d) and interfollicular region (if), the majority of cells within the follicular remants (f), and cells scattered throughout the muscularis (ms). *b*: The stain for macrophages (Du2-66) revealed scattered cells (solid triangles) in the dome region (d) and many cells (solid triangles) scattered throughout the follicular remnants (f). *c*: The stain for CD4$^+$ T cells revealed cells scattered throughout the lamina propria, interfollicular region (if), dome (d), and rare cells (solid triangles) within the follicular remnant (f). *d*: The stain for B cells (CD45R) revealed numerous cells within the dome (d), few cells in the interfollicular region (if), and rare cells (solid triangles) surviving within the follicular remnant (f). Magnification: *a–d* (bar [in *d*] = 100 μm).

provided a sensitive method to detect B-cell production in ileal PP follicles. There was no detectable BrdU incorporation in the ileal PP follicular remnants for 3 weeks after dexamethasone treatment (Figs. 2h and 2i). IHC analysis of 10–20 serial sections did not reveal BrdU incorporation in the follicles despite consistent BrdU incorporation in mucosal crypt epithelium and cells in the interfollicular region. Thus, dexamethasone induced a complete arrest of iPfB-cell proliferation, but small foci of proliferating iPfB-cells reappeared between 28–35 days postdexamethasone (Fig. 4a) and the size of this proliferating population increased during the following 2 months (Figs 4b and 4c). However, the regenerating follicles were heterogeneous in size and misshapen when compared with ileal PP follicles from age-matched control lambs (Fig. 2g) and there was no further increase in follicular size between days 54–98 posttreatment (Fig. 4c). BrdU incorporation in normal jejunal PPs revealed a high level of cell proliferation in mucosal crypt epithelium and the lymphoid follicles, but few cells proliferating in the dome or interfollicular area (Fig. 2d). Dexamethasone treatment did not inhibit cell proliferation in the crypt epithelium or the lymphoid follicles despite a marked reduction in follicle size (Fig. 2e). Following dexamethasone treatment, the jejunal PP follicles rapidly increased in size and

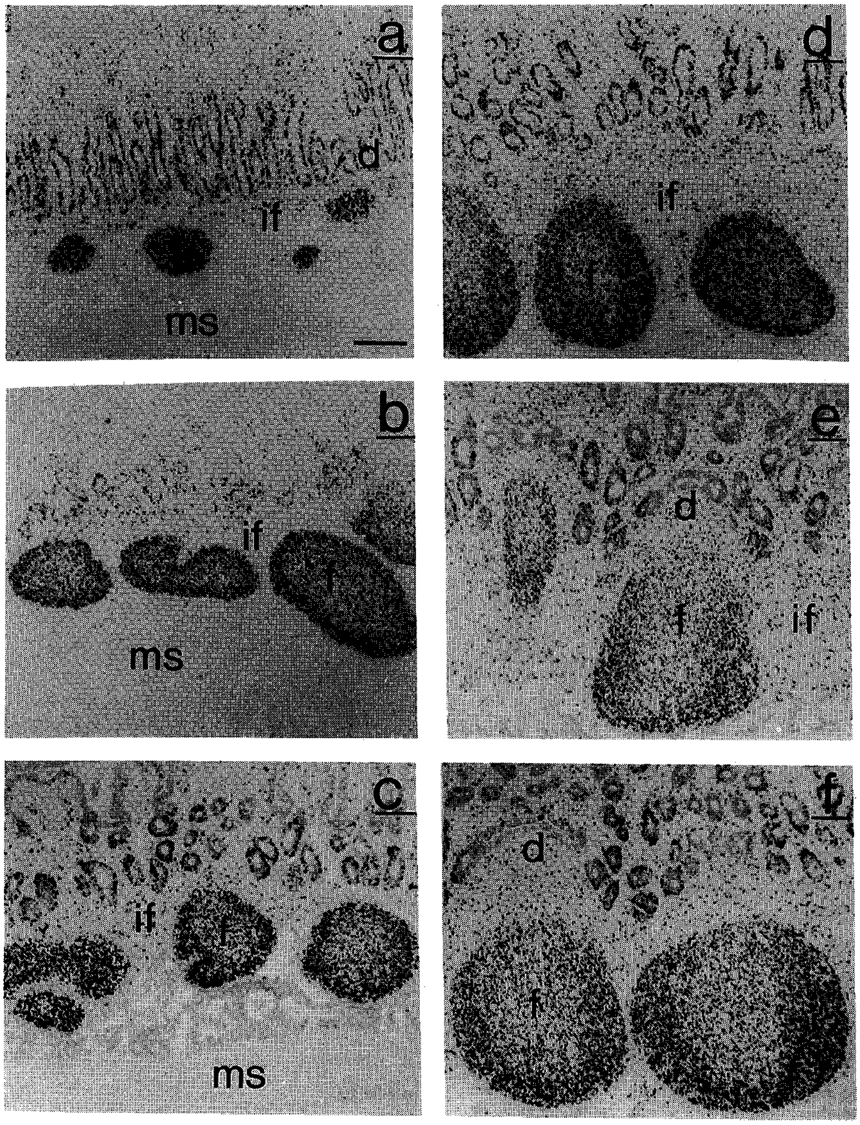

FIGURE 4. Cell proliferation, detected by BrdU incorporation, in the ileal and jejunal PPs following treatment for 7 days with 2 mg dexamethasone/kg BW (postdex). Ileal PP: *a* (D.32—postdex); *b* (D.54—postdex); and *c*: (D.98—postdex). BrdU incorporation was consistently detected in villus crypt epithelium and scattered cells in the interfollicular region (if). Foci of proliferating cells were detected in follicles (f) on D.32 posttreatment, but the size of this proliferating population did not increase beyond D.54 posttreatment. The muscularis (ms) is indicated beneath the follicles. Jejunal PPs: *d* (D.32—postdex); *e* (D.54—postdex); and *f*: (D.98—postdex). A high level of BrdU incorporation was evident within the lymphoid follicles (f) at all times posttreatment and was associated with a gradual increase in follicle size. The pattern of BrdU incorporation in the villus crypt epithelium, interfollicular region (if), and dome region (d) remained constant. Magnification: *a*–*f* (bar [in *a*] = 350 μm).

cellularity (Figs 2f, 4d, e and f) until they were similar to follicles in untreated lambs (Fig. 2d).

### B-cell Phenotype and Function in Regenerating PPs

The marked variation in follicle size in the regenerating ileal PP (Figs 4a, b, and c) suggested that an altered B-cell population was developing within the follicles. Therefore, we examined the normal variation in ileal and jejunal PP follicular size using a method to release intact follicles from PPs of young lambs (Griebel and Ferrari, 1995) and by examining serial tissue sections of PPs from a 144-day fetal lamb injected for 1 hr with 20 mg BrdU/kg BW. From these analyses, it was apparent that lymphoid follicles in the ileal and jejunal PPs of untreated, 6–10-week-old lambs (Figs 5a and 5b) and a fetal lamb (Figs 5c and 5d) varied 4–5-fold in size and also varied in shape. Similar size and shape variation was observed with both histology and mechanically isolated follicles and the variation in follicle size occurred despite cell proliferation in all fetal follicles (Figs 5c and 5d). Thus, variable follicle size in the regenerating ileal PP may reflect the normal variation in PP follicular size and the process(es) responsible for variable follicle size during fetal development. The phenotype of cells isolated from

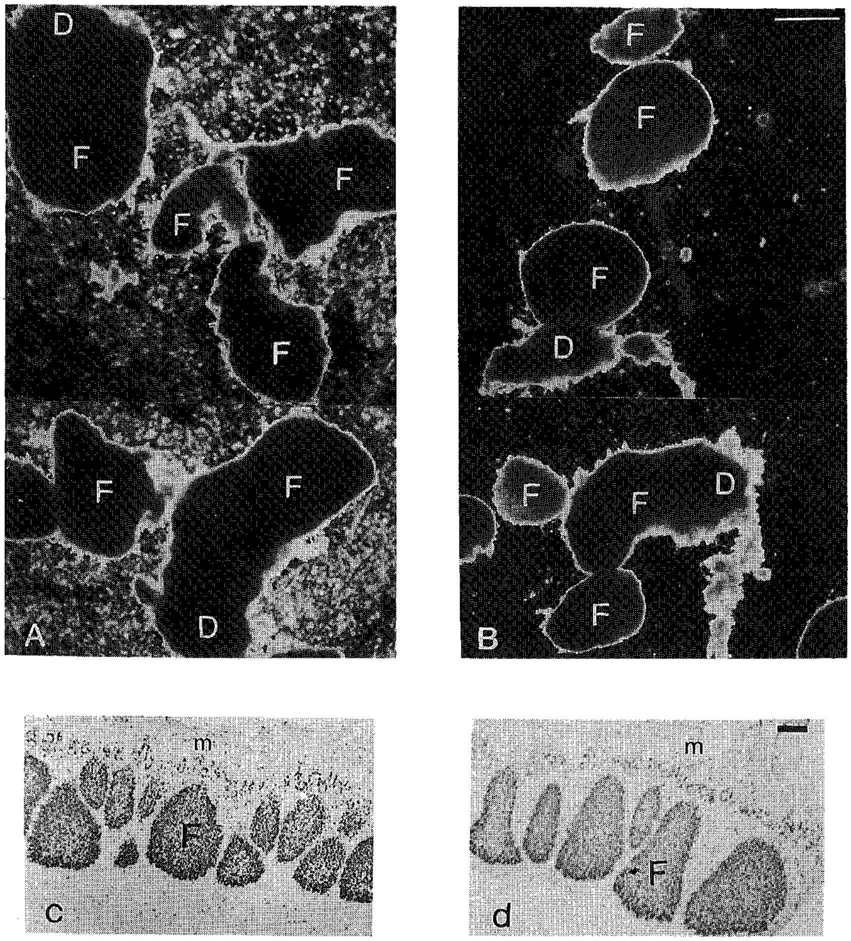

FIGURE 5. Lymphoid follicles in the PPs of foetuses and young lambs varied markedly in size. A: Lymphoid follicles (F) mechanically isolated from the ileal PP of a 6-week-old lamb display considerable variation in size and shape that was independent of the presence or absence of an attached dome region (D). Follicles viewed with phase-contrast microscopy. B: Lymphoid follicles (F) isolated from the jejunal PPs display a similar variation in size and shape independent of an attached dome region (D). Follicles viewed with phase-contrast microscopy. C: All follicles (F) in the fetal (144-day gestation) ileal PP had incorporated BrdU but varied in size. BrdU incorporation was also evident in the crypt epithelium of the mucosa (m). These observations were based on the examination of 45 serial tissue sections stained for BrdU. d: Fetal jejunal PP displayed consistent BrdU incorporation within follicles (F) and crypt epithelium (m) but a marked variation in follicular size and shape. These observations were based on the examination of 38 serial tissue sections stained for BrdU. Magnification: A, B (bar [in B] = 500 μm); magnification: c, d (bar [in d] = 100 μM).

regenerating ileal and jejunal PP follicles was also analyzed. These analyses did not reveal significant differences between cells in regenerating follicles and cells isolated from PP follicles of untreated age-matched controls (Table 1).

To evaluated if dexamethasone treatment altered B-cell function, we examined the production of IgA and IgG1 PCs in the PPs. The jejunal PPs contain numerous IgA PCs in the lamina propria adjacent to villus crypts, in the interfollicular region, the dome

TABLE 1
Phenotype of Lymphocytes Isolated from Lymphoid Follicles of the Ileal and Jejunal PPs

| Phenotype | Ileal PP follicles[a] | | Jejunal PP follicles[a] | |
|---|---|---|---|---|
| | Control[b] | Postdexamethasone | Control | Postdexamethasone |
| CD40[d] | 63.2 ± 6.4 | 74.4 ± 7.7 | 53.7 ± 7.2 | 47.8 ± 6.1 |
| sIgM | 93.5 ± 2.8 | 88.4 ± 4.0 | 44.6 ± 7.4 | 35.0 ± 6.6 |
| $CD5^{lo}sIgM^{+}$ [e] | 2.5 ± 1.8 | 3.8 ± 1.9 | 28.9 ± 8.6 | 31.2 ± 6.9 |
| sIgG1 | 2.7 ± 1.3 | 4.4 ± 1.6 | 20.5 ± 8.4 | 24.8 ± 7.3 |
| sIgA | 0.3 ± 0.3 | 0.9 ± 1.0 | 16.3 ± 7.8 | 22.0 ± 6.2 |
| $CD44^{hi}$B-cells[f] | 9.0 ± 1.5 | 9.6 ± 1.8 | 2.7 ± 1.2 | 2.8 ± 0.8 |
| $CD4^{+}$ T-cells | 0.42 ± 0.2 | 0.33 ± 0.08 | 14.8 ± 5.3 | 16.3 ± 6.1 |

[a] FACS analyses of cell suspensions prepared from lymphoid follicles. [b] Data presented are the mean ± S.D. of values from 5, 6–12-week-old lambs. [c]Data presented are the mean ± S.D. of values for 6 lambs analyzed between days 42–98 posttreatment. Data were pooled after analyses showed no significant differences. [d] The CD40 molecule was detected using soluble mouse CD40 ligand-CD8α detected with PE-conjugated rat anti-mouse CD8α. [e] Values are the % $sIgM^{+}$ B cells that coexpressed CD5. [f] The B-cell population was defined by subtracting $CD5^{hi}$ cells (T-cells) from the total $CD44^{hi}$ population. The light-scatter gates used for data collection excluded macrophages and stromal cells from the analyses.

region, and within the follicle (Fig. 6d). IgA PCs are absent in ileal PP follicles, but otherwise have a distribution similar to the jejunal PPs (Fig. 6a). Following dexamethasone treatment, there was no change in the distribution or apparent frequency of IgA PCs in either the ileal (Figs. 6b and 6c) or jejunal PPs (Figs. 6e and 6f; Table 1). Similar observations were made for IgG1 PCs that were located primarily in the dome region, in the follicles, and in the interfollicular regions of the jejunal PPs and within

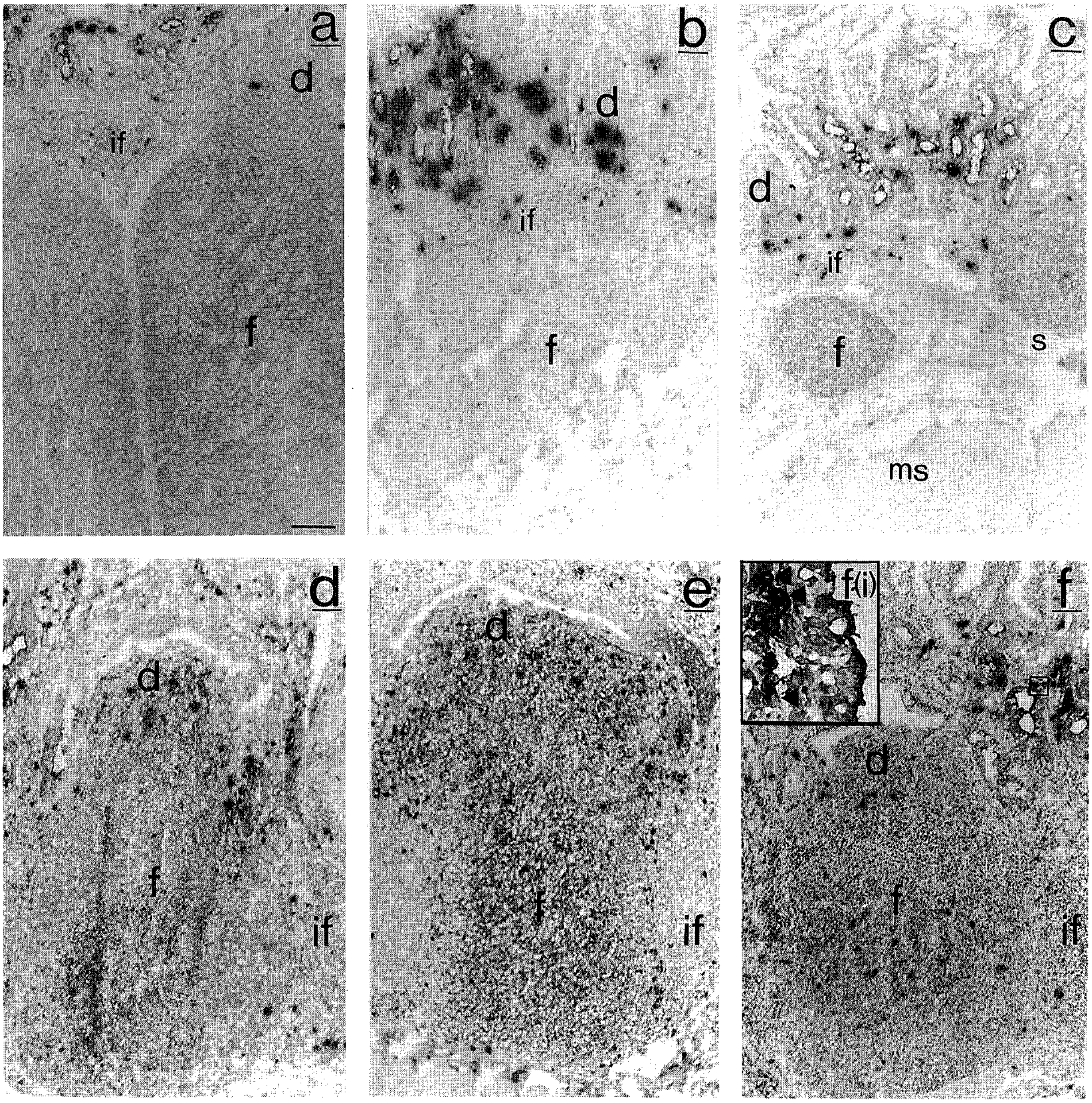

FIGURE 6. Immunoperoxidase staining for IgA revealed a similar distribution of IgA PCs in the PPs in age-matched, untreated lambs (control) and lambs treated for 7 days with 2 mg dexamethasone/kg BW (postdex), Ileal PP: **a** (control); **b** (D.21—postdex); **c** (D.74—postdex). IgA PCs in the ileal PP were located primarily in the lamina propria, adjacent to the villus crypt, and rare PCs are seen in the interfollicular (if) and dome (d) region but never in follicles (f). Jejunal PPs: **d** (control); **e** (D.21—postdex); **f** (D.74—postdex). IgA PCs in the jejunal PP were numerous in the lamina propria, adjacent to the villus crypt, and numerous in the dome region (d), in the follicle (f), and the interfollicular region (if). **f***(i) inset*: Higher magnification of area outlined in **f** shows intense cytoplasmic staining of PCs in the lamina propria and surface staining of the mucosal epithelium. Magnification: **a–f** (bar [in **a**] = 150 μm).

the dome and interfollicular region of the ileal PP (data not shown; Table 1). The observations on follicle structure, B-cell phenotype, and PP function indicated that the distinct functions performed by ileal and jejunal PPs remained intact following dexamethasone treatment.

### Depletion of T Lymphocytes in Blood

An acute depletion of greater than 50% blood T-cells began during and progressed for 1 week following dexamethasone treatment (Fig. 7b). This T lymphopenia involved all T-cell subsets and persisted for approximately 6 weeks. The recovery of circulating T-cell numbers to pretreatment levels was influenced most by an increase in the predominant γδ TcR-cell population. This occurred between 6–8 weeks posttreatment and followed thymic regeneration by approximately 3 weeks (Fig. 7). Flow cytometric analysis of CD44 expression on $CD5^{hi}$ cells (T-cells) indicated that thymic involution and regeneration were linked to changes in blood T-cell populations. The subpopulation of $CD44^{lo}CD5^{hi}$ cells disappeared during dexamethasone treatment (Fig. 8; day 01 postdexamethasone) and did not reappear until 6 weeks posttreatment (Fig. 8; day 40 postdexamethasone). The reappearance of $CD44^{lo}CD5^{hi}$ cells followed thymic regeneration (Fig. 7a) and preceded the recovery of circulating T-cells (Fig. 7b). Thus, the temporal order of these events was consistent with the thymus regenerating the circulating T-cell population.

### Depletion of B Lymphocytes in Blood

Dexamethasone treatment induced an acute and prolonged decline in blood B-cell numbers and the composition of this B-cell population was markedly changed (Fig. 9). In young lambs, approximately 90% blood B-cells ($CD40^+$) express sIgM (Fig. 9a) and less than 10% $sIgM^+$B cells express detectable levels of CD5 (Fig. 8; pretreatment). One week after dexamethasone treatment less than 20% $sIgM^+$B cells remained and >90% B cells expressed CD5 (Fig. 8; day 01 postdexamethasone). Furthermore, the relative contribution of isotype-switched B cells to the total B-cell population increased to 50% following dexamethasone treatment (Figs. 9a and 9b). The number of circulating $sIgG1^+$ and $sIgA^+$ B cells remained relatively constant following dexamethasone treatment, but these B-cell populations varied widely among individual animals (Fig. 8b).

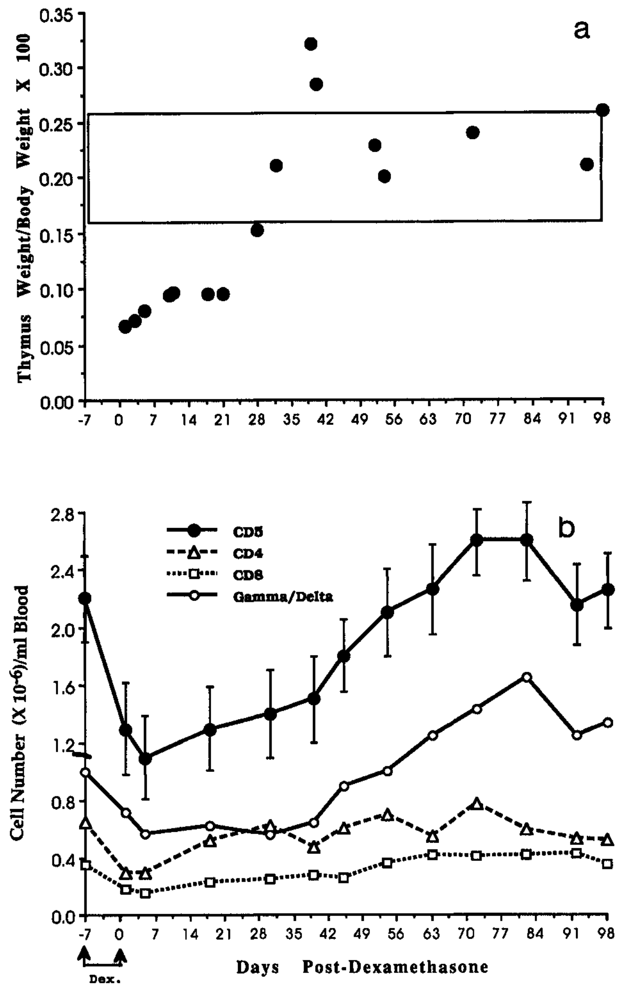

FIGURE 7. Dexamethasone treatment (dex) induced a marked decrease in thymic weight and the number of T lymphocytes in blood. a: Thymic weights were 20–35% of the normal weight immediately after dexamethasone treatment (day 0), but 4–5 weeks later there was a complete recovery of thymic mass. Each data point represents the weight of a single thymus, as a percentage of BW, and the range of normal thymic weights is depicted by the rectangle. b: The total number of T lymphocytes ($CD5^{hi}$) and all T-lymphocyte subsets (CD4, CD8, γδ TcR [gamma/delta]) declined markedly following dexamethasone treatment. The total number of T lymphocytes returned to pretreatment levels 8 weeks after dexamethasone treatment with γδ T lymphocytes contributing the most to the T-lymphocyte population. Data presented are the mean ± S.D. of values from varying numbers of lambs (days −7 and 0: $n$ = 16; day 98: $n$ = 2).

Specificity of the ST1a mAb for CD5 on B cells was assessed in two ways. First, $CD5^{hi}Du2\text{-}104^-$ cells and $CD5^{lo}Du2\text{-}104^+$ cells were sorted with a FACStar cell sorter and cytospots prepared of the two populations. IHC analyses of cytospots revealed that $CD5^{hi}Du2\text{-}104^-$ were 98% $CD3^+$ (T-cells) with no detectable staining for IgM or Ig LC. In contrast,

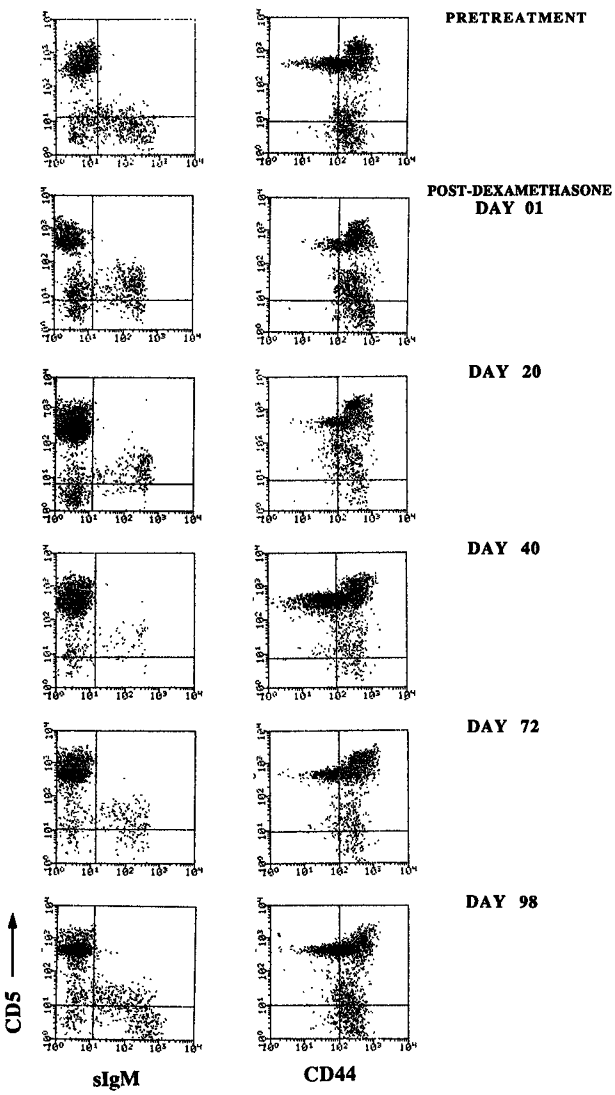

FIGURE 8. Phenotypic analysis of B and T lymphocytes present in blood before and after dexamethasone treatment (2 mg/kg for 7 days). In young lambs (pretreatment), few B-cells expressed detectable levels of CD5, but immediately following dexamethasone treatment (postdexamethasone: day 01), most $sIgM^+$B-cells expressed a low level of CD5. The predominance of $CD5^+IgM^+$ B-cells persisted for 14 weeks posttreatment (postdexamethasone day 98) with a minor subpopulation of $CD5^-sIgM^+$B-cells. The changes in $CD5^+IgM^+$B-cell number were reflected in the $CD5^{lo}CD44^{hi}$ population. In young lambs (pretreatment), T-cells ($CD5^{hi}$) were clearly divided into $CD44^{lo}$ (32.4 + 3.8% of T-cells) and $CD44^{hi}$ subpopulations (66.9 + 5.7% of T-cells). During dexamethasone treatment, the $CD44^{lo}CD5^{hi}$ subpopulation (postdexamethasone day 01) was markedly reduced (7.4 + 1.9% of T-cells) and this reduction persisted for at least 3 weeks (postdexamethasone day 20). However, 6 weeks after dexamethasone treatment (postdexamethasone day 40), the relative frequency of the $CD44^{lo}CD5^{hi}$ subpopulation had recovered (38.6 + 4.7% of T-cells) to a level comparable to pretreatment lambs. The X axis presents sIgM expression (column 1) or CD44 expression (column 2) and the Y-axis presents CD5 expression for the dot scatter plots.

$CD5^{lo}Du2\text{-}104^+$ cells were 2% $CD3^+$, 83% $sIgM^+$, and 95% Ig $LC^+$. Second, the STla mAb immunoprecipitated a 67-kD protein from iPfB-cells cocultured with J558L cells transfected with murine CD40 ligand, but no protein was immunoprecipitated from normal iPfB-cells (data not shown). Few iPfB-cells bind STla mAb (Table 1), but 60–70% iPfB-cells bind a detectable level of the STla mAb following coculture with CD40 ligand (Griebel and Ferrari, 1995). Thus, present experiments indicate that in blood, the most steroid-sensitive population of B-cells were $CD5^-sIgM^+$ and, in the absence of a functional ileal PP, this population remained severely depleted. A small number of $CD5^-sIgM^+$ B-cells were evident between 9–10 weeks posttreatment and this population increased a little during the next 4 weeks (Fig. 8; day 98 postdexamethasone; Fig. 9a). Thus, a return in iPfB-cell proliferation was followed 4 weeks later by the appearance of a limited number of $CD5^-sIgM^+$ B-cells in blood (Figs. 4a and 9a). The prolonged B lymphopaenia was in marked contrast to the recovery in circulating T-cell number (Fig. 8). Finally, a transient neutrophilia during dexamethasone treatment was the only alteration in blood polymorphonuclear leukocyte, monocyte, and red blood cell populations observed throughout these experiments (data not shown). These observations, together with thymic regeneration, indicate that hematopoiesis in bone marrow was not altered by dexamethasone. Thus, it seems unlikely that altered B lymphopoiesis in the bone marrow could explain the prolonged B lymphopenia in blood.

### DISCUSSION

The present experiments clearly demonstrated that, as expected for a population of immature lymphocytes, the B-cells in lymphoid follicles of the ileal PP were very corticosteroid-sensitive (Fig. 1). Dexamethasone treatment completely disrupted the function of the ileal PP (Fig. 2h), but had relatively minor effects on the jejunal PP (Figs. 2e, 2f, 6e and 6f). Extensive follicular involution in the ileal PP after treatment with dexamethasone for 72 hr (data not shown) was consistent with a direct cytolytic effect on iPfB-cells. However, it remains to be determined if dexamethasone also acted indirectly by disrupting signals that supported iPfB-cell growth despite the survival of many mesenchymal cells in the follicular remnants (Fig. 3). Clearly, corticosteroid treatment inhibited much more effectively the T-cell–independent proliferation of iPfB-cells than the T-cell–dependent development of B-cells in the jejunal PPs (Griebel and Ferrari, 1994; Griebel and Ferrari, 1995). Thus, B-cell development in these two PP was shown to be distinct.

The absence of detectable iPfB-cell proliferation for a 4-week period did not result in a permanent arrest of iPfB-cell development (Fig. 4). Thus, sustained self-renewing proliferation is not essential to maintain the iPfB-cell population as was previously suggested by *in vitro* experiments (Griebel and Ferrari, 1994). The involuted follicles may have been repopulated by B-cells surviving within the ileal PP follicles or dome region (Fig. 3d) or B-cells circulating in the blood (Fig. 9). If either intrafollicular or dome region B-cells were the source of the proliferating iPfB-cells, this would imply the existence of a functionally distinct, steroid-resistant stem B-cell that can give rise to the rapidly dividing, steroid-sensitive iPfB-cells. Alternatively, it could be postulated that, as for the thymus, the bone marrow functions as the primary source of B-cell progenitors that then undergo further development in the unique microenvironment of the ileal PP follicles. The present experiments could not determine the source of B-cells in regenerating ileal PP follicles. However, it was evident that the regenerating ileal PP maintained the unique function of this tissue. This conclusion was supported by the analyses of follicle structure (Fig. 5), B-cell phenotype (Table 1), and PP function (Fig. 6). Finally, the limited regeneration of ileal PP follicles (Fig. 4c) may also reflect normal ileal PP development because ileal PP involution usually begins at 3–4 months of age (Reynolds and Morris, 1983).

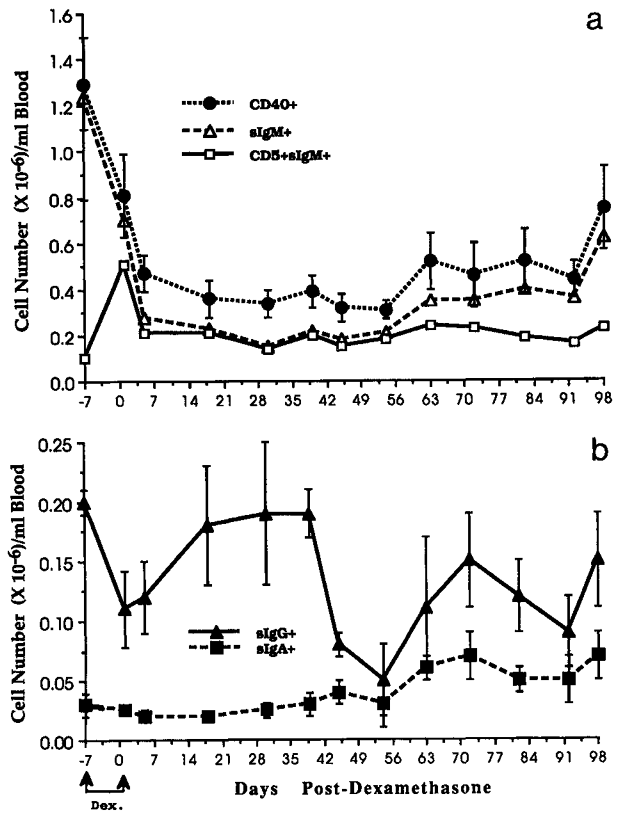

FIGURE 9. There was a sharp decline in total B-cell numbers in blood following dexamethasone treatment (2 mg/kg for 7 days) and this B lymphopenia persisted throughout the experiment. a: One week following dexamethasone treatment, the total number of B-cells (CD40$^+$) in blood had declined to 30% of pretreatment values. The sIgM$^+$ B-cells (sIgM$^+$) accounted for 90% of B-cells in blood prior to dexamethasone treatment, but less than 50% following dexamethasone treatment. Finally, CD5$^+$sIgM$^+$B-cells (CD5$^+$sIgM$^+$) were a minor population in young lambs, but more than 90% sIgM$^+$ B-cells expressed a detectable level of CD5 following dexamethasone treatment. A detectable level of CD5$^-$ sIgM$^+$B-cells were apparent 80 days posttreatment and then gradually increased. b: Dexamethasone treatment did not significantly alter the number of sIgG1$^+$B-cells (sIgG$^+$) or sIgA$^+$B-cells (sIgA$^+$) in blood. Considerable variation in isotype-switched B-cell number was noted within and among experimental groups. Following dexamethasone treatment, the isotype-switched B-cells comprised 50% of the total B-cell population. Data presented are the mean ± S.D. of values from varying numbers of lambs (days −7 and 0: $n$ = 16; day 98: $n$ = 2; see Fig. 7a).

It is difficult to explain the prolonged quiescent period in B-cell production if the ileal PP follicles were regenerated by intrafollicular B-cells. The detection of BrdU incorporation in individual mucosal epithelial cells and interfollicular cells (Figs. 2h and 2i) indicated that limited sensitivity of BrdU detection could not explain the absence of proliferating iPfB-cells. Alternatively, despite the survival of follicular architecture (Fig. 3), the dexamethasone treatment may have disrupted nonlymphoid stromal-cell functions that support iPfB-cell proliferation (Griebel and Ferrari, 1994). Dexamethasone can inhibit the production of diverse cytokines (Bettens et al., 1984; Culpepper and Lee, 1985; Lee et al., 1988; Waage and Bakke, 1988) and alter the functional state of a wide variety of cells (Cupps and Fauci, 1982). Thus, if time were required to regenerate functional stromal cells, then it may have limited iPfB-cell proliferation. Alternatively, the delay in ileal PP regeneration may also be explained by the time required for sIgM$^+$ B-cell differentiation in bone marrow and then the homing of naive B-cells to the ileal PP. An analysis of the frequency of Ig λ V gene somatic mutation (Reynaud et al., 1995) in B-cells of regenerated ileal PP follicles may reveal the source of stem B-cells if the assumption applies that bone-marrow-derived B-cells would be unmutated and an intrafollicular stem cell was mutated prior to dexamethasone treatment. Alternatively, transplantation of involuted ileal PP follicles into SCID mice may reveal intrafollicular stem B-cells if all follicular elements essential for the support of iPfB-cell proliferation survived dexamethasone treatment and the immigration of stromal-cell elements was not integral to PP regeneration.

Previous experiments, using surgical extirpation of the ileal PP in fetal and new-born lambs, indicated that the ileal PP was the major source of B-cells in young lambs (Reynolds et al., 1985; Gerber et al., 1986). This implied that bone marrow played a minor role in B lymphopoiesis or that the ileal PP provided an essential environment for B-cell development. The effects of dexamethasone-induced involution of the ileal PP were similar to surgical extirpation (Gerber et al., 1986) with both a severe and prolonged B lymphopenia (Fig. 9) and no disruption of gut-associated IgA PC production (Table 1: Fig. 6). The present experiments clearly indicated that in the absence of a functional ileal PP, there were no other sources of circulating $CD5^-sIgM^+$ B-cells (Fig. 9a) and the resumption of a reduced level of iPfB-cell proliferation (Fig. 4a) was followed by the reappearance of a small number of $CD5^-sIgM^+$ B-cells in blood (Fig. 9a). The B lymphopaenia observed 98 days postdexamethasone was even more dramatic when compared to the number of B-cells ($2.5–3.0 \times 10^6$ cells/ml blood) reported for 4–5-month-old lambs (Hein et al., 1990). Thus, the low level of iPfB-cell proliferation in the regenerating ileal PP was not capable of effectively generating circulating $CD5^-sIgM^+$ B-cells. In contrast, the number of $CD5^+sIgM^+$ B-cells remained relatively constant despite the presence or absence of a functional ileal PP (Fig. 9a), indicating that the generation or maintenance of $CD5^+$ B-cells was not linked to the ileal PP. This is consistent with the paucity of $CD5^+sIgM^+$ B-cells in the ileal PP and the presence of numerous $CD5^+sIgM^+$ B-cells in the jejunal PP (Table 1). Thus, as suggested by *in vitro* experiments, the expression of CD5 on sheep B-cells may simply be an indication of T-cell–dependent B-cell activation (Griebel and Ferrari, 1995). The persistence of $CD5^+$ B-cells in sheep would be consistent with observations in mice and humans, which indicated that corticosteroid treatment does not prevent secondary, T-cell–dependent, B-cell responses (Cupps and Fauci, 1982).

A profound depletion of circulating B-cells (>90%) and T-cells was also observed during prolonged dexamethasone treatment of young calves (Oldham and Howard, 1992). In these experiments, a similar dosage of dexamethasone was used and the resulting B lymphopaenia indicates that corticosteroid sensitivity may be characteristic for ileal PP B-cells in young ruminants and possibly other species where this lymphoid tissue is prominent. Consistent with this conclusion is the observation that the age of an animal is an important factor in determining the effect of dexamethasone treatment on blood lymphocytes populations (Oldham and Howard, 1992). From the present experiments, it is clear that dexamethasone treatment can disrupt lymphocyte production in both the thymus and ileal PP (Fig. 1) and marked changes in the circulating lymphocyte populations follow as a natural consequence (Fig. 8). Thus, the present model of dexamethasone-induced involution of the ileal PP may provide a means to identify in which species the ileal PP functions as a primary source of circulating B-cells. Finally, previous *in vitro* observations were used to define various species as either corticosteroid-sensitive or corticosteroid-resistant (Clamen et al., 1971), but these definitions have ignored the fact that B-cell development may vary significantly in young animals of different species.

In conclusion, the present experiments clearly demonstrated that ileal PP follicles contain a population of steroid-sensitive B-cells and there may also be an intrafollicular steroid-resistant B-stem-cell population. In young lambs, the ileal PP was essential for the generation of $CD5^-sIgM^+$ B-cells, but T-cell–dependent B-cell development can continue in other lymphoid tissues. Furthermore, during the absence of a functional ileal PP $CD5^+sIgM^+$ B-cell numbers remain constant (Fig. 9). Thus, dexamethasone-induced involution of the ileal PP may provide a good model with which to study humoral immune responses when the "conventional" or $CD5^-$ B-cell population is severely depleted. Also, it may be possible to determine if sheep $CD5^+sIgM^+$ B-cells represent, as in mice (Kantor and Herzenberg, 1993), a functionally distinct lineage of B-cells or B-cells that have responded to T-cell–dependent antigens (Griebel and Ferrari, 1995). If the latter is true, then primary humoral immune responses may be compromized in the absence of $CD5^-sIgM^+$ B-cells, but secondary humoral immune responses should remain intact. Experiments are in progress to test this hypothesis and determine if dexamethasone involution of the ileal PP is a simple method to produce lambs with a humoral immune deficiency.

### ACKNOWLEDGMENTS

We gratefully acknowledge the assistance of Mark Dessing and Stefan Meyer with cell sorting and thank Drs. W.R. Hein, B.A. Imhof, and J.-C. Weill for critical review of the manuscript. The Basel Institute for Immunology was founded and is supported by Hoffman La Roche and Company, Basel, Switzerland.

*(Received May 30, 1995)*

*(Accepted July 3, 1995)*

### REFERENCES

Abe K., and Ito T. (1977). A qualitative and quantitative morphologic study of Peyer's patches of the mouse. Arch. Histol. Jpn. **40**: 407–420.

Bettens F., Kristensen F., Walker C., Schawulera U., Bonnard G.D., and de Weck A.L. (1984) Lymphokine regulation of activated (G1) lymphocytes. II. Glucocorticoid and anti-Tac-induced inhibition of human T lymphocyte proliferation. J. Immunol. **141**: 1191–1196.

Beya M.-F., Miyasaki M., Dudler L., Ezaki T., and Trnka Z. (1986). Studies on the differentiation of T lymphocytes in sheep. II. Two monoclonal antibodies that recognise all ovine T lymphocytes. Immunology **57**: 115–121.

Carlens O. (1928). Studien ueber das lymphatische Gewebe das Darmkanals bei einegin Haustieren, mit besonderer Beruecksichtigung der embryonalen Entwicklung, der Mengenverhaeltnisse und der Altersinvolution dieses Gewebes im Duenndarm des Rindes. Z. Anat. Entwicklungsgesch **86**: 393–493.

Chapman H.A., Johnson J.S., and Cooper M.D. (1974). Ontogeny of Peyer's patches and immunoglobulin-containing cells in pigs. J. Immunol. **112**: 555–563.

Claman H.N., Chaperon E.A., and Triplett R.F. (1966). Thymus-marrow cell combinations: Synergisms in antibody production. Proc. Soc. Exp. Biol. Med. **122**: 1167–1171.

Clamen H.N., Moorhead J.W., and Brenner W.H. (1971). Corticosteroids and lymphoid cells *in vitro*. I. Hydrocortisone lysis of human, guinea pig, and mouse thymic cells. J. Lab. Clin. Med. **78**: 499–507.

Cornes J.S. (1965). Number, size, and distribution of Peyer's patches in the human small intestine. Part I. The development of the Peyer's patches. Gut **6**: 225–233.

Craig S.W., and Cebra J.J. (1971). Peyer's patches: An enriched source of precursors for IgA-producing immunocytes in the rabbit. J. Exp. Med. **134**: 188–200.

Culpepper J.A., and Lee F. (1985). Regulation of IL-3 expression by glucocorticoids in cloned murine T lymphocytes. J. Immunol. **135**: 3191–3197.

Cupps T.R., and Fauci A.S. (1982). Corticosteroid-mediated immunoregulation in man. Immunol. Rev. **65**: 133–155.

Ezaki T., Miyasaka M., Beya M.-F., Dudler L., and Trnka Z. (1987). A murine anti-sheep T8 monoclonal antibody, ST-8, that defines the cytotoxic T lymphocyte population. Int. Archs. Allergy Appl. Immunol. **82**: 168–177.

Gerber H.A., Morris B., and Trevella W. (1986). The role of gut-associated lymphoid tissues in the generation of immunoglobulin-bearing lymphocytes in sheep. Aust. J. Exp. Biol. Med. Sci. **64**: 201–213.

Glick B. (1957). Experimental modification of the growth of the bursa of Fabricius. Poultry Science **36**: 18–23.

Griebel P.J., and Ferrari G. (1995). CD40 signalling in ileal Peyer's patch B cells: Implications for T cell-dependent antigen selection. Int. Immunol. **7**: 369–379.

Griebel P.J., and Ferrari G. (1994). Evidence for a stromal cell-dependent, self-renewing B cell population in lymphoid follicles of the ileal Peyer's patch of sheep. Eur. J. Immunol. **24**: 401–409.

Griebel P.J., Kennedy L., Graham T., Davis W.C., and Reynolds J.D. (1992). Characterisation of B cell phenotypic changes during ileal and jejunal Peyer's patch development in sheep. Immunology **7**: 564–570.

Hein W.R., Dudler L., and Mackay C.R. (1989). Surface expression of differentiation antigens on lymphocytes in the ileal and jejunal Peyer's patches of lambs. Immunology **68**: 365–370.

Hein W.R., Dudler L., and Morris B. (1990). Differential peripheral expansion and *in vivo* antigen reactivity of α/β and γ/δ T cells emigrating from the early foetal lamb thymus. Eur. J. Immunol. **20**: 1805–1813.

Hummel K.P. (1935). The structure and development of the lymphatic tissue in the intestine of the albino rat. Amer. J. Anat. **57**: 351–377.

Husband A.J., and Gowans J.L. (1978). The origin and antigen-dependent distribution of IgA-containing cells in the intestine. J. Exp. Med. **148**: 1146–1160.

Ishidate M., and Metcalf D. (1963). The pattern of lymphopoiesis in the mouse thymus after cortisone administration or adrenalectomy. Aust. J. Exp. Biol. **41**: 637–649.

Kantor A.B., and Herzenberg L.A. (1993). The origin of murine B lineages. Annu. Rev. Immunol. **11**: 501–538.

Lane P., Brocker T., Hubele S., Lanzavecchi A., and McConnel F. (1993). Soluble CD40-ligand can replace normal T cell derived CD40-ligand signal to B cells in T-dependent activation. J. Exp. Med. **177**: 1209–1213.

Lee S.W., Tsou A.-P., Chan H., Thomas J., Petrie K., Eugui E.M., and Allison A.C. (1988). Glucocorticoids selectively inhibit the transcription of the interleukin 1β gene and decrease the stability of interleukin 1β mRNA. Proc. Natl. Acad. Sci. USA **85**: 1204–1208.

Mackay C.R., Beya M.-F., and Matzinger P. (1989). γδ T cells express a unique surface molecule appearing late during thymic development. Eur. J. Immunol. **19**: 1477–1483.

Mackay C.R., Maddox J.F., and Brandon M.R. (1987). A monoclonal antibody to the p220 component of sheep LCA identifies B cells and a unique lymphocyte subset. Cell. Immunol. **110**: 46–55.

Mackay C.R., Maddox J.F., Wijffels G.L., Mackay I.R., and Walker I.D. (1988). Characterisation of a 95,000 molecule on sheep leukocytes homologous to murine Pgp-1 and human CD44. Immunology **65**: 93–99.

Maddox J.F., Mackay C.R., and Brandon M.R. (1985). Surface antigens SBU-T4 and SBU-T8 of sheep lymphocyte subsets defined by monoclonal antibodies. Immunology **55**: 739–749.

Mitchell G.F., and Miller J.F.A.P. (1968). Cell to cell interaction in the immune response. II. The source of hemolysin-forming cells in irradiated mice given bone marrow or thymus or thoracic duct lymphocytes. J. Exp. Med. **128**: 821–837.

Oldham G., and Howard C.J. (1992). Suppression of bovine lymphocyte responses to mitogens following *in vivo* and *in vitro* treatment with dexamethasone. Vet. Immunol. Immunopath. **39**: 161–177.

Osmond D.G. (1980). The contribution of the bone marrow to the economy of the lymphoid system. Monogr. Allergy **16**: 157–172.

Pollard M., and Sharon N. (1970). Responses of the Peyer's patches in germ-free mice to antigenic stimulation. Infect. Immunity **2**: 96–100.

Press C. McL., Hein W.R., and Landsverk T. (1993). Ontogeny of leucocyte populations in the spleen of foetal lambs with emphasis on the early prominence of B cells. Immunology **80**: 598–604.

Reynaud C.-A., Garcia C., Hein W.R., and Weill J.-C. (1995). Hypermutation generating the sheep immunoglobulin repertoire is an antigen-independent process. Cell **80**: 115–125.

Reynaud C.-A., Mackay C.R., Muller R.G., and Weill J.-C. (1991). Somatic generation of diversity in a mammalian primary lymphoid organ: the sheep ileal Peyer's patches. Cell **64**: 995–1005.

Reynolds J.D. (1986). Evidence of extensive lymphocyte death in sheep Peyer's patches. I. A comparison of lymphocyte production and export. J. Immunol. **136**: 2005–2010.

Reynolds J.D. (1987). Mitotic rate maturation in the Peyer's patches of foetal sheep and in the bursa of Fabricius of the chick embryo. Eur. J. Immunol. **17**: 503–507.

Reynolds J.D., Kennedy L., Peppard J., and Pabst R. (1991). Ileal Peyer's patch emigrants are predominantly B cells and travel to all lymphoid tissues in sheep. Eur. J. Immunol. **21**: 283–289.

Reynolds J.D., and Morris B. (1983). The evolution and involution of Peyer's patches in foetal and postnatal sheep. Eur. J. Immunol. **13**: 627–635.

Reynolds J.D., Pabst R., and Bordmann G. (1985). Evidence for the existence of two distinct types of Peyer's patches in sheep. *In*: Microenvironments in the lymphoid system, Klaus G.G.B., Ed. (London: Plenum), pp. 101–109.

Waage A., and Bakke O. (1988). Glucocorticoids suppress the production of tumour necrosis factors by lipopolysaccharide-stimulated human monocytes. Immunology **63**: 299–302.

Waksman B.H., Ozer H., and Blythman H.E. (1973). Appendix and γM-antibody formation. IV. The functional anatomy of the rabbit appendix. Lab. Invest. **28**: 614–626.

